# A Case of Membranous Croup, with Complete Laryngeal Cast

**Published:** 1880-01

**Authors:** 


A Case of Membranous Croup, with Complete Laryngeal Cast.—In the evening of July 31st, 1879, I was called to see Anna S., aged eight years, living in this village. The little girl had been complaining for two or three days, and was at first supposed by the parents to be suffering with common cold. When I first saw her the general symptoms were not alarming. There was but little disturbance of the circulation; no febrile movement. The breathing was at times labored, both in inspiration and expiration. There were no laryngeal spasms, so frequent in false croup. There was no evidence of exudation within the pharynx. As I have said, there were no symptoms which especially denoted gravity; yet the fact of the disturbance of respiration, together with a peculiar change in the voice, with an occasional, short, barking, croupal cough, led me to fear an exudation within the larynx.

The next morning, August 1st, I again saw the patient. There was now no doubt as to the trouble. The obstruction had increased; the breathing was more labored; the voice was a husky whisper; the face was congested; there was some febrile movement; there was distressing dyspœa. Examining some of the matter expectorated, I found membraniform patches, making the diagnosis certain. Laryngitis with exudation existed, or membranous croup.

The patient died on the morning of August 3d. Surgical interference was suggested as a possible means of relief, but the parents decided not to permit an operation. The treatment consisted in supporting measures largely. During August 1st the heart began to fail. Quinia, milk, animal broths, and whisky, were freely used.

I call attention to this case simply to mention the occurrence, during the progress of it, of an event quite infrequent in membraneous croup, namely, the detachment of a complete cast of the larynx. Patches of membrane frequently occur in this disease, being found in the expectorated matter, and I have seen fibrinous casts of the bronchial tubes; but I have now in my possession a cast of the whole larynx, as it was coughed up by this child on the morning of August 2d. Immediate relief followed for twenty-four hours. Of course the membrane was reproduced and the patient died on the morning of August 3d.

I may speak of the measures which, as I believe, contributed largely to the promotion of the suppurative process by which the false membrane was separated in this instance. I applied a blister of the acetic cantharidal vesicant over the larynx, and enveloped the neck for a time in a poultice, afterwards making continuous application of cold by means of compresses dipped in ice water, applied and removed at intervals, after the manner advocated by Prof. Peaslee.—*Ohio Med. Recorder.*

